# Use of a Closure Device for the Management of Inadvertent Placement of a Central Venous Catheter in the Carotid Artery: A Case Report and Literature Review

**DOI:** 10.7759/cureus.34911

**Published:** 2023-02-13

**Authors:** Ioakeim Giagtzidis, Andrea Soteriou, Christina Papadimitriou, Ioakeim Papoutsis, Christos Karkos

**Affiliations:** 1 5th Surgical Department/Vascular Surgery, Hippokrateio General Hospital/Aristotle University of Thessaloniki, Thessaloniki, GRC

**Keywords:** central venous catheter (cvc), catheter-related complications, direct carotid artery puncture, percutaneous arterial closure device, inadvertent puncture

## Abstract

The placement of a central venous catheter (CVC) is a common intervention in hospitalized patients. Several adverse events have been reported in this “blind” procedure when it is performed without the aid of ultrasound, including artery catheterization, which although uncommon, is a serious complication. Potential treatment options include manual compression, open surgical repair, and endovascular treatment. A 62-year-old critically ill patient with accidental arterial catheterization of the right common carotid artery (CCA) during placement of CVC is presented. The catheter was removed successfully with the use of a Perclose-ProGlide closure device. A systematic literature review was performed to identify similar cases treated with the same technique. This case presents an alternative minimally invasive treatment option, using a Perclose Proglide (Abbott) closure device for the removal of a misplaced CVC in the right CCA. Although this is an off-label use of the device it can be an effective alternative treatment option, especially in unstable patients.

## Introduction

Central venous access is a common procedure providing necessary vascular access. Its implementation increased proportionally, especially during the pandemic, where approximately 25% of COVID-19 patients required access through a central vein [1].

There are many complications associated with central venous catheterization which can lead to a prolonged hospital stay, increased costs for the healthcare system, and decreased quality of life [1]. Specifically, complications of internal jugular vein cannulation include infection, embolism, thrombosis, arrhythmias, hematoma, pneumothorax, cardiac perforation and tamponade, fistula formation, and arterial and nerve injury [1]. To reduce or minimize the incidence of these complications, the ultrasound-guided puncture is nowadays mandatory [2]; however, inadvertent arterial puncture during central venous catheter (CVC) placement occurs in 2%-4.5%, causing arterial injury in 0.1%-0.5% of patients [2]. Specifically, when access involves the jugular vein, incidental arterial puncture is reported between 6.3% and 9.4% of the cases with the incidence of arterial cannulation being around 1% [3].

Several case reports and case series have described different treatment strategies for injury to the aortic arch, subclavian, brachiocephalic, or carotid artery during the attempted placement of jugular or subclavian venous catheters. Manual compression can be used but it can lead to devastating complications such as hematoma, stroke, and death especially if the catheter is 7Fr and above [4]. Traditionally open surgical repair has been the treatment of choice, however endovascular surgery with the use of covered stents is gaining popularity due to its minimally invasive approach and easier access to difficult anatomic areas [4]. Since endovascular procedures are becoming the standard of care and they continue to evolve, several closure devices have been available to achieve hemostasis after the percutaneous approach [4]. Although not described in their instructions for use (IFU), there have been few reports of the use of these devices, for treating accidental arterial placement of CVCs. This study is a case report of using a Perclose-ProGlide (Abbott Vascular Inc., Santa Clara, CA, USA) closure device to remove a mispositioned CVC in the right CCA, and review of the relevant literature.

## Case presentation

**Figure 1:**
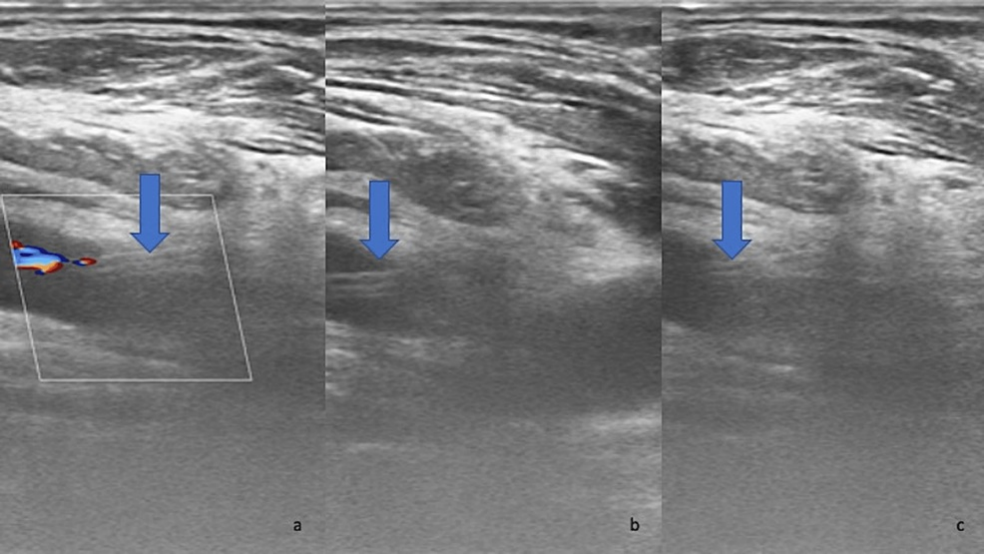
Duplex ultrasound identifying the misplaced catheter

A 62-year-old male came to the ER with signs of acute respiratory failure triggered by a lower respiratory infection. The patient had a past medical history of atrial fibrillation (AF), hypertension, cardiac failure, and morbid obesity. A few hours after his arrival, he became unstable, so he was intubated and transferred to the ICU. During attempted catheterization of his right internal jugular vein, a triple lumen 7Fr CVC was accidentally placed in his right common carotid artery (CCA). Initial clinical suspicion of inadvertent placement of the catheter was raised from pulsating back-bleeding. A bedside duplex ultrasound was performed by a vascular surgeon where the misplaced catheter was identified in the right CCA (Figures 1a-1c).

**Figure 2:**
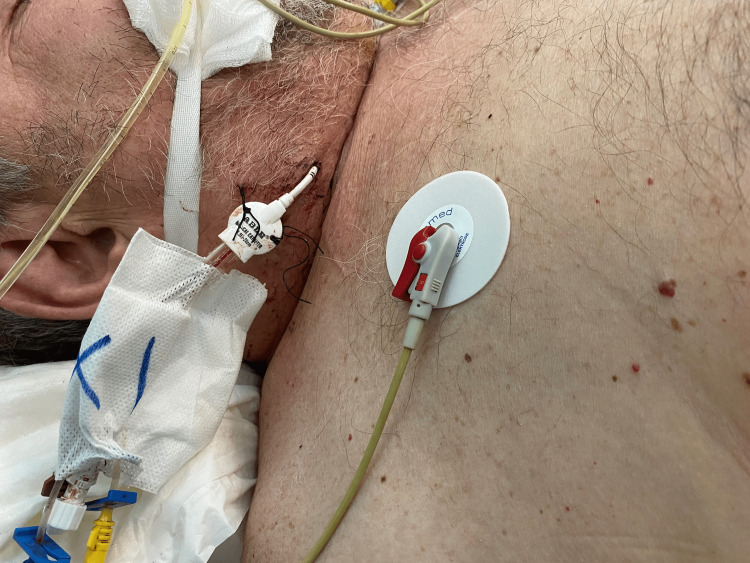
Hostile wide and short neck

**Figure 3:**
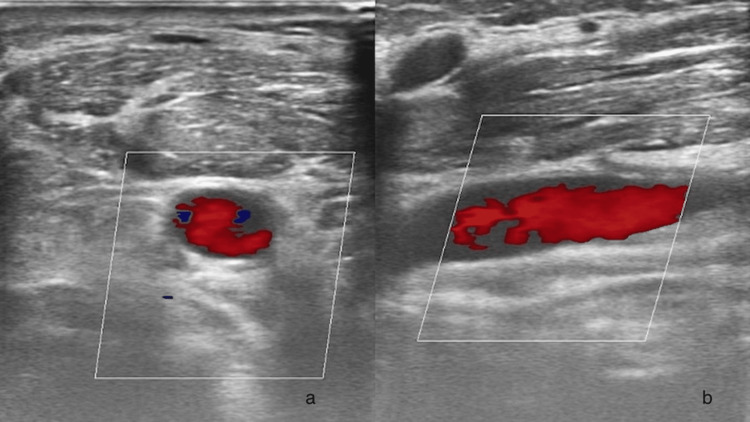
Duplex ultrasound verifying hemostasis after use of the closure device

Because of the patient’s severe condition and short wide neck (Figure 2), he was transferred to the operating room, where it was decided to remove the CVC with the use of a Perclose-ProGlide system. A hydrophilic stiff guidewire 0.035’’-180cm was introduced through the distal lumen of the CVC and it was removed over the wire with manual compression. A Perclose-ProGlide device was advanced over the wire and successfully deployed according to IFU. Hemostasis was achieved and verified with ultrasound on the table (Figures 3a, 3b), through the absence of direct flow outside the CCA. Νo further manual compression was required, and the patient returned to ICU. No complications were identified postoperatively.

A review was performed using the PubMed database between 2000 and 2022. The following terms were used: “central venous catheter” OR “central line” AND “closure device”. Inclusion criteria included any case reports or case series with misplaced CVC in the carotid artery, treated with any percutaneous closure device. The search returned 72 results. Each article was independently reviewed along with their references, to verify that the injured artery was the carotid and to extract the number of patients treated, the size of the CVC, the closure device that was used and the results. Any misplaced catheter in an anatomic area other than the CCA, or in the carotid artery treated with no use of a closure device was excluded from the study.

Overall, eight publications with 15 patients described accidental cannulation of their carotid artery with a CVC and treated with a closure device (Table 1) [5-12]. In six cases (40%) the inserted catheter was 7Fr, in another six cases (40%) was 8.5Fr, in one case (6.6%) it was 9Fr in another one 6Fr (6.6%) and in one last case (6.6%) it was 12Fr. Regarding the devices that were used, Perclose-Proglide was used in the majority (n=8, 53.3%) of the cases. Two patients (n=2, 13.3%) were managed with Boomerang (Cardiva Medical, Mountain View, California) and two more (n=2, 13.3%) with Exoseal (Cordis Corporation, Bridgewater, NJ). In the final three cases one (n=1, 6.6%) Mynx (AccessClosure, Mountain View, CA) one (n=1, 6.6%) StarClose (Abbott Vascular, Santa Clara, CA) and one (n=1, 6.6%%) AngioSeal (St. Jude Medical, St. Paul, MI) were deployed. An embolic protection device (Spider FX, Medtronic, Dublin, Ireland) was used in just one case were the AngioSeal was placed.

Overall technical success was 93.3% (n=14). In one patient with a 9FR catheter misplaced in the common carotid, the ExoSeal could not achieve complete sealing, so a secondary endovascular intervention was performed with the deployment of a stent graft (Fluency 10x40mm, BARD Medical, Covington, GA).

**Table 1:** Cases of carotid artery injury treated with closure devices. n=number of patients, Fr=French

Study	Year	Patients (n)	Catheter size (Fr)	Symptoms	Closure Device	Complications
Yoon et al [5]	2015	1	7	No	Mynx	None
Pikwer et al [6]	2009	1	12	No	Perclose	None
Kirkwood et al [7]	2008	2	7	No	Boomerang	None
Stellmes et al [8]	2014	2	7,9	N/A	Exoseal	None, Exoseal failed secondary Fluency stent graft
Bechara et al [9]	2014	6	8.5	N/A	Percose	None
Gandhi et al [10]	2016	1	7	No	Angioseal	None
Lorenzo et al [11]	2020	1	7	No	Perclose	None
Pua et al [12]	2015	1	6	No	StarClose	None

## Discussion

Insertion of a CVC or a dialysis catheter is a common procedure in hospitalized, surgical, and renal patients for the administration of medications, intravenous fluids, parental nutrition, hemodialysis, and monitoring. In the United States, more than 5 million CVCs are placed annually [13]. Despite the efforts to avoid adverse events, mainly with the recommendation of ultrasound guidance, their placement is associated with infectious, thromboembolic, and mechanical complications increasing significantly in-hospital morbidity and mortality [14]. Complications of arterial puncture and cannulation of a CVC, include bleeding, hematoma, pseudoaneurysm, dissection, arterio-venous fistula formation, arrhythmia, stroke, and even death [14].

Several factors have been associated with mechanical complications such as obesity or very low BMI, hypotension, duration of the procedure, and insertion of a CVC in an emergency setting or during the night [15]. Furthermore, adverse events are related to the male gender, and the number of punctures per attempt, when more than two punctures can lead to up to 54% of failure or mechanical complication [15]. Interestingly regarding the insertion site, there is some evidence that there are more arterial punctures (3.0% vs 0.5%) but less catheter malpositions (5.3% vs 9.3%) in the internal jugular compared with the subclavian access [16].

Early detection of an arterial injury during CVC placement is of great importance and can prevent more catastrophic complications [15]. If there is clinical or laboratory suspicion of a misplaced catheter, this should be confirmed with any imaging modality, and the catheter should be left in place [15,17]. Withdrawal of a catheter located in the carotid artery and manual compression should also be avoided since it may result in hemorrhage, hematoma, airway obstruction, stroke, or pseudoaneurysm formation [17].

Open surgical repair is safe and seems to be the standard of care but can add to morbidity since these patients are already critically ill and require general anesthesia [17]. Furthermore, these operations add a significant workload to any vascular unit and anesthesiology department. Technical advancement and collective experience in endovascular procedures offer a safe alternative for managing misplaced catheters. Different endovascular options, like balloon tamponade, tract embolization and covered stent provide excellent technical success with a minimally invasive approach and local anesthesia [18]; however, balloon tamponade might be inadequate and in case of stent graft use, long-term patency and lifelong use of antiplatelets or anticoagulation might be an issue [18].

Another endovascular solution that can be offered is the use of closure devices. The exponential growth of endovascular procedures created the need for an alternative or additional method for managing postprocedural access sites besides manual compression. In 1995 Vasoseal (St Jude Medical, St. Paul, MN) was the first FDA-approved vascular closure device and over the past 25 years, several other devices have become commercially available [19]. Overall, these devices can be divided into three categories. The active ones physically close the arteriotomy with sutures or clips, the passive ones deploy a plug of some kind on the arteriotomy site, and one last category promotes coagulation externally via patches or pads with clotting factors [19]. Overall, their use is shortening the time to hemostasis, patient ambulation, and discharge while according to Boghal et al., their complication rate is non-inferior to manual compression [19]. Despite their extensive utilization, their IFUs still include only the common femoral artery. However, there have been numerous publications on the successful use of closure devices in many off-label locations such as superficial and profunda femoral artery, brachial, subclavian, and carotid artery following percutaneous endovascular procedures [19]. Furthermore, in a recent systematic review of the management all inadvertent arterial placement of CVCs, the authors suggest the use of percutaneous closure devices as the first line approach in case femoral, subclavian, brachial, and carotid artery is involved [20].

## Conclusions

The use of CVCs in everyday medical practice is increased especially during the Covid-19 pandemic, resulting in an increased number of iatrogenic vascular injuries. CVC placement especially in the internal jugular vein should be performed under ultrasound guidance. Even if all necessary precautions are taken, arterial and especially carotid injury and accidental catheterization can lead to devastating complications.

Percutaneous closure devices although widely used in endovascular procedures, are not frequently utilized as a treatment option for inadvertent placement of CVC especially when the carotid artery is involved. This is probably due to their strict IFU, and the learning curve experience needed for their successful deployment. They seem to be however a safe, quick, minimally invasive, and effective treatment option for misplaced catheters in frail or unstable patients, that can be performed even bedside. Several publications suggest that these devices should be used as first-line treatment in such cases but in order to be widely implemented and not presented as merely case reports and small case series, further comparative studies should be designed between several devices and treatment options.

This report examines the use of percutaneous closure devices as a treatment option for the inadvertent placement of CVCs, especially in the carotid artery. Including the patient in this case report, only 16 patients treated with the same technique can be found in the literature. The use of these devices provides many advantages, that justify their wider and more frequent use.
